# Gravity-Dependent Modulation of Downbeat Nystagmus and Subjective Visual Vertical in the Roll Plane

**DOI:** 10.1007/s12311-024-01685-y

**Published:** 2024-03-18

**Authors:** Stefan Macher, Daniela Dunkler, Anuscha Theresa Fiehl, Paulus Stefan Rommer, Kirsten Platho-Elwischger, Felix Konstantin Schwarz, Gerald Wiest

**Affiliations:** 1 Department of Neurology, Medical University of Vienna, Vienna, Austria; 2 Center for Medical Data Science, Institute of Clinical Biometrics, Medical University of Vienna, Vienna, Austria

**Keywords:** Downbeat nystagmus, Subjective visual vertical, Otolith, Graviceptive function

## Abstract

Downbeat nystagmus (DBN) is the most common form of acquired central vestibular nystagmus. Gravity perception in patients with DBN has previously been investigated by means of subjective visual straight ahead (SVA) and subjective visual vertical (SVV) in the pitch and roll planes only during whole-body tilts. To our knowledge, the effect of head tilt in the roll plane on the SVV and on DBN has not yet been systematically studied in patients. In this study, we investigated static and dynamic graviceptive function in the roll-plane in patients with DBN (patients) and healthy-controls (controls) by assessment of the Subjective Visual Vertical (SVV) and the modulation of slow-phase-velocity (SPV) of DBN. SPV of DBN and SVV were tested at different head-on trunk-tilt positions in the roll-plane (0°,30° clockwise (cw) and 30° counterclockwise (ccw)) in 26 patients suffering from DBN and 13 controls. In patients, SPV of DBN did not show significant modulations at different head-tilt angles in the roll-plane. SVV ratings did not differ significantly between DBN patients vs. controls, however patients with DBN exhibited a higher variability in mean SVV estimates than controls. Our results show that the DBN does not exhibit any modulation in the roll-plane, in contrast to the pitch-plane. Furthermore, patients with DBN show a higher uncertainty in the perception of verticality in the roll-plane in form of a higher variability of responses.

**Supplementary Information:**

The online version contains supplementary material available at 10.1007/s12311-024-01685-y.

## Introduction

Patients with downbeat nystagmus (DBN) typically report that almost any movement with deflection from the vertical leads to an increase in oscillopsia and imbalance. Gravity-dependent modulations of ocular drift in the pitch plane was accordingly found in these patients [1–3]. So far, there exist only contradictory data on the modulation of DBN in the roll-plane during whole-body tilts [2, 3]. Studies at the perceptual level can also provide information about the pathophysiology of the imbalance in these patients. Gravity perception of DBN patients has previously been investigated by assessment of the subjective visual straight ahead (SVA) and in the pitch and roll plane only during whole-body tilts [1, 4, 5]. In the latter, the perception of verticality is based not only on altered afferences from the otolith system, but also from the somatosensory system [4]. In order to ensure almost exclusive otolith stimulation, we investigated the modulation of DBN and SVV in the roll-plane during static head-tilt, which, to our knowledge, has not yet been studied at the ocular motor and perceptual level.

## Objectives

The aim of the study was to investigate to what extent the SPV of DBN and SVV is altered in patients by changing the head position in the roll plane. Based on previous findings of patients with DBN who were exposed to whole body tilts in the roll plane, we hypothesised that the SPV of DBN is not altered by isolated changes in head position in the roll plane, but that the SVV estimates of the patients differ significantly from those of normal controls in the roll plane. Based on this hypothesis, we have formulated the following primary objectives: 1) measurement of SPV alterations during CW (30°, right) and CCW (-30°, left) head-tilts in the roll plane in patients and 2) determining whether SVV-estimates differ between patients and controls at 0° head position, at 30° CW, and at 30° CCW head-tilts in the roll plane.

## Methods

### Subjects

In this prospectively planned study, twenty-six consecutive patients (24 patients with idiopathic DBN, 2 patients with pontine lesions) diagnosed with downbeat-nystagmus were recruited from the Neurotology Outpatient Clinic, Department of Neurology, Medical University of Vienna. (Sample size calculation can be found in supplemental methods). All patients received neurological examination, cranial MRI-scan, Video-oculography and rotational testing and genetic testing if applicable. The SVV-estimates of thirteen healthy subjects served as controls. This study was approved by the ethical committee of the Medical University of Vienna.

## Recording Methods

Ocular motor and vestibular function was assessed using binocular video-oculography (sampling rate 60 Hz) and a computer-controlled rotational chair system (System 2000, Micromedical Technologies, Illinois, USA,). A plastic head ring and a hook and loop tape that was mounted on an adjustable neck rest (which covered the occiput and the posterior neck) fixed the head. The interval between each test/head position was approximately 3 min. This experimental setup was used in a previous study [6].

## Assessment of Downbeat-Nystagmus

DBN was assessed by a single measurement at each of the three different head positions: 1) head fixed upright (0°), 2) at head tilt angle of 30° to the left (CCW) and 3) at head tilt angle of 30° to the right (CW). Slow-phase velocity (SPV, deg/sec) of DBN was determined by computer-controlled analysis.

## Assessment of SVV

Patients were seated in a dark room in front of a rotatable dim light bar (10 cm length). The bar was adjusted 6 times from randomized starting positions for alignment with the perceived gravitational vertical in each of the following head positions: fixed upright (0°), 30° CCW and CW head tilt.

## Statistics

While continuous variables are reported as median and 1. and 3. quartiles, categorical variables are reported as absolute numbers and percentages. For each participant, the mean of the six repeated SVV measures was used. To answer the two primary objectives, linear mixed models with a random effect for the participant were estimated. The log-transformed SPV was used in the linear mixed model. However, reported means are calculated for the original SPV data. Independent variables were defined as head tilt angle (-30°, 0°, 30°) and additionally for the SVV model DBN (patients, controls). The variance–covariance matrix was selected optimizing the Akaike information criterion. The two-tailed alpha was set at 0.05. Analyses were performed in SAS 9.4 (SAS Institute, Cary NC) and R 4.2.2 (R Core Team 2022).

## Results

All 26 patients completed this prospective cross-sectional cohort study. Patients were median 72 years old (44 – 86 years, 62% [n = 16] male). Median disease duration was 2.25 years (0.5 – 10 years). Except for 4-Aminopyridine and other CNS-active-agents, co-medication was unchanged during the study period (n = 11). On the day of the examination, the patients were not taking any CNS active medication. Controls were median 52 years old (33 – 72 years, 69.2% [n = 9] female**).**

### SPV Modulation in Patients

**Fig. 1:**
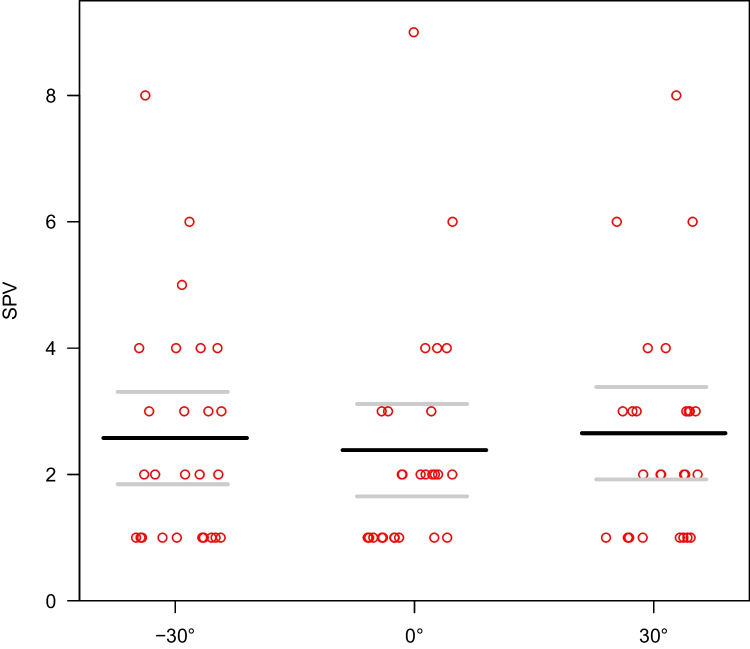
A linear mixed model of SPV (deg/sec) with tilt angle (-30°, 0°, 30°) in patients with DBN. Least-squares means and 95% confidence intervals are shown as black and gray lines

A linear mixed model of SPV with angle (-30°, 0°, 30°) in patients only found a non-significant effect of angles (p = 0.108) (Fig. 1). Mean SPV values were 2.58 deg/s at -30° (CI 1.85–3.31), 2.38 deg/s at 0° (CI 1.65–3.12) and 2.65 deg/s at 30° (CI 1.92–3.39).

### SVV in Patients and Controls

**Fig. 2:**
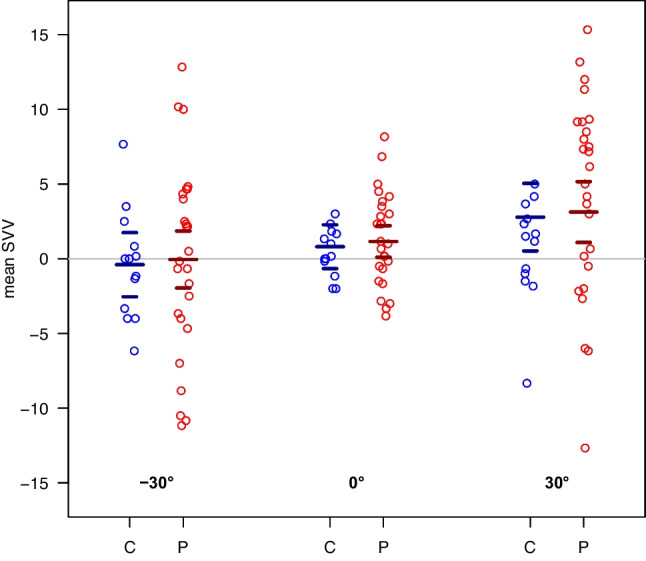
A linear mixed model of the mean SVV with tilt angle, DBN and its pair-wise interaction showed no statistically significant interaction (p = 0.243). Hence, least-squares means from a main effects model with 95%-CI are shown. (C = controls, P = patients)

A linear mixed model of the mean SVV with DBN (patients versus controls), angle (-30°, 0°, 30°) and its pair-wise interaction showed no statistically significant interaction (p = 0.243) (Fig. 2). Observed differences in mean SVV between patients and controls were also medically not relevant. Hence, we estimated a main effects model including DBN and angle only. We found a statistical significant difference between angles (p = 0.021), but not for DBN (p = 0.695) (Supplement Table 1).

Interestingly, mean SVV ratings showed a greater variance in patients than in controls at all head tilt angles, which was most striking at 30° (Fig. 2).

## Discussion

It is well established that the SPV of DBN exhibits a gravity dependent modulation in the pitch plane [3, 7, 8]. Several studies using whole body rotation along this plane, showing that SPV is lowest around the supine and highest in the prone body position [1–3]. Recent studies suggest that the modulation of SPV along the pitch plane can be best explained by deficient input–output coupling of gravitational otolith-input related to control of vertical eye position (1). Regarding the gravity dependent modulation of downbeat nystagmus in the roll-plane, only contradictory data exist so far. Modulation of nystagmus was described in a patient with DBN during static whole-body tilts in the pitch and roll plane (2). Another study, which also applied whole-body tilts, found no modulation of vertical drift velocity in 6 patients with DBN in the roll-plane (3). By using almost pure otolithic stimulation with head-tilts—even if a certain somatosensory input from neck receptor cannot be completely ruled out [9]—we could not observe a medically relevant and statistically significant modulation of SPV of DBN in the roll-plane in 26 patients. This finding is in line with a previous study using whole-body tilts [3]. This obvious dissociation between a gravity dependence of DBN along the pitch plane but not the roll plane implies that there is no disinhibited otolith-ocular reflex in patients with DBN in the roll-plane. While patients with DBN exhibit a directional upward-bias in their adjustments of the SVA [5] we could not observe a significant difference in the SVV adjustments between patients and controls in the roll-plane, in particular there was no significant directional bias. However, all patients with DBN exhibited a higher variability in their SVV adjustments at 0° and at all head tilts, which was most impressive at 30°. A higher variability in verticality perception has previously also been shown in DBN patients using whole-body tilts in the roll plane (1). This phenomenon may in our opinion not be attributed to oscillopsia, as SPV of DBN was very low in our patients. Furthermore, the lack of limb ataxia in our patients also rules out a motor problem as a cause. The mean age of participants of our control group was lower than that of our patients, however, a previous study in healthy individuals has shown that age does not influence SVV responses [10].

## Conclusion

Our results suggest, that the increased SVV errors in our patients are due to a disturbed central estimate of gravitational direction, caused by vestibulocerebellar dysfunction [1]. Our findings imply that altered otolith input in the roll-plane changes the verticality of patients with DBN at the perceptual but not at the ocular motor level.

## Supplementary Information

Below is the link to the electronic supplementary material.

Supplementary file1 (DOCX 22 KB)

## Data Availability

Data available on reasonable request from the authors.
