# Supplementary material for: Gravity-Dependent Modulation of Downbeat Nystagmus and Subjective Visual Vertical in the Roll Plane
Source: Cerebellum. 2024 Mar 18;23(5):1899–902. doi: 10.1007/s12311-024-01685-y (PMC11489239; doi:10.1007/s12311-024-01685-y)
Supplement: Supplementary file 1 — Supplementary file1 (DOCX 22 KB) [file 12311_2024_1685_MOESM1_ESM.docx]

**Supplement**

Supplemental methods: *Sample size calculation*

We have reserved a significance-level of 0.025 for each of the two primary research

questions. Based on the data of the control patients from Elwischger et al (2017), the

mean absolute SVV 0° measurement is 1.3° with a standard deviation of 1, for the

mean of the mean absolute SVV 30° measurements (left and right) side the mean is

2.7° with a standard deviation of 1.5. For the patients, it is expected that the

variability of the measurements will be larger, we assumed an increase of 50%,

leading to a standard deviation of 1.5 for 0° measurements and 2.25 for 30°

measurements. A mean change of 2° at each measurement, 0° and 30°, is assumed

to be clinically relevant. No drop out is expected, as the complete examination will be

completed within 30 minutes. For the 30° measurement, a larger sample size is required: A two group Satterthwaite t-test with a 0.025 two-sided significance level will have 82% power to detect a difference in means of -2 (the difference between a group 1 mean of 2.7 and a group 2 of 4.7) assuming that the group 1 standard deviation, is 1.5 and the group 2 standard deviation is 2.25 (ratio of group 2 to group 1 standard deviation is 1.5) when the sample sizes in the two groups are 13 and 26, respectively. Hence, measurement of 26 patients (2:1 matching with the already

available controls) will be obtained.

| **Effect** | **category** | **LSMEANS or**  **differences of LSMEANS** | **adjusted p-value** |
| --- | --- | --- | --- |
| **angle** | ***-30°*** | *-0.22(-2.07; 1.62)* |  |
|  | ***0°*** | *0.98 (0.054; 1.91)* |  |
|  | ***30°*** | *2.96 (0.99; 4.93)* |  |
|  | **30° vs. -30°** | 3.18 (0.27; 6.09) | 0.029 |
|  | **30° vs. 0°** | 1.97 (0.17; 3.78) | 0.029 |
|  | **-30° vs. 0°** | -1.21 (-3.46; 1.05) | 0.404 |
| **DBN** | ***patients*** | *1.41 (0.12; 2.70)* |  |
|  | ***controls*** | *1.06 (-0.57; 2.70)* |  |
|  | **patients vs. controls** | 0.35 (-1.42; 2.11) | 0.695 |

Supplement Table 1: A linear mixed model of the mean SVV with tilt angle (-30°, 0°, 30°) and DBN (patients, controls). Least-squares means (italic letters) and differences of least-squares means with 95% confidence intervals of the mean SVV are reported.
